# Real-World Data on Alcohol Consumption Behavior Among Smartphone Health Care App Users in Japan: Retrospective Study

**DOI:** 10.2196/57084

**Published:** 2025-03-25

**Authors:** Kana Eguchi, Takeaki Kubota, Tomoyoshi Koyanagi, Manabu Muto

**Affiliations:** 1 Department of Informatics Graduate School of Informatics Kyoto University Kyoto Japan; 2 Department of Real World Data Research and Development Graduate School of Medicine Kyoto University Kyoto Japan; 3 Business Development Office Institute for Advancement of Clinical and Translational Science (iACT) Kyoto University Hospital Kyoto Japan; 4 Department of Therapeutic Oncology Graduate School of Medicine Kyoto University Kyoto Japan

**Keywords:** alcohol consumption, individual behavior, mobile health, mobile health app, mobile health care app log-based survey, real-world data, RWD, RWD analysis, smartphone health care app, surveillance system, health care app

## Abstract

**Background:**

Although many studies have used smartphone apps to examine alcohol consumption, none have clearly delineated long-term (>1 year) consumption among the general population.

**Objective:**

The objective of our study is to elucidate in detail the alcohol consumption behavior of alcohol drinkers in Japan using individual real-world data. During the state of emergency associated with the COVID-19 outbreak, the government requested that people restrict social gatherings and stay at home, so we hypothesize that alcohol consumption among Japanese working people decreased during this period due to the decrease in occasions for alcohol consumption. This analysis was only possible with individual real-world data. We also aimed to clarify the effects of digital interventions based on notifications about daily alcohol consumption.

**Methods:**

We conducted a retrospective study targeting 5-year log data from January 1, 2018, to December 31, 2022, obtained from a commercial smartphone health care app (CALO mama Plus). First, to investigate the possible size of the real-world data, we investigated the rate of active users of this commercial smartphone app. Second, to validate the individual real-world data recorded in the app, we compared individual real-world data from 9991 randomly selected users with government-provided open data on the number of daily confirmed COVID-19 cases in Japan and with nationwide alcohol consumption data. To clarify the effects of digital interventions, we investigated the relationship between 2 types of notification records (ie, “good” and “bad”) and a 3-day daily alcohol consumption log following the notification. The protocol of this retrospective study was approved by the Ethics Committee of the Kyoto University Graduate School and Faculty of Medicine (R4699).

## Introduction

Alcohol consumption contributes to 3 million deaths globally each year, with harmful use responsible for 4.7% of the global burden of disease [1]. The World Health Organization recommends reducing harmful alcohol use by developing surveillance systems for alcohol consumption [2]. Although several studies using smartphone apps have been conducted [3], none have examined the general population’s long-term alcohol consumption behavior, that is, for a period of time longer than a year, nor the effect of digital interventions.

To elucidate detailed alcohol consumption behavior among alcohol drinkers in Japan, we conducted a retrospective study targeting 5-year logs obtained from a commercial smartphone health care app (CALO mama Plus [4]; Wellmira Inc). Common drinking occasions for Japanese working people include welcome events, get-togethers, farewell parties, and regular after-work drinking parties, so we hypothesized that there was a decrease in alcohol consumption during the period of the COVID-19 outbreak and the state of emergency, when individuals were requested to stay home and reduce social gatherings. We tested this hypothesis with individual real-world data; this analysis was only possible with such data. We aimed to clarify the effects of digital interventions using notifications and daily records of alcohol consumption.

## Methods

### Overview

We analyzed the smartphone app logs to investigate two perspectives: (1) user numbers and (2) alcohol consumption behavior (ie, mean net alcohol consumption and alcohol-related notifications, together with their effectiveness). Because our target period coincided with the COVID-19 outbreak, we compared these values with the daily number of confirmed COVID-19 cases in Japan [5].

### Target Commercial Smartphone Health Care App

CALO mama Plus [4] is a mobile health care app for managing nutrition. It records various health-related data, including alcohol consumption, and has been used in several health-related studies based on real-world data [6-9]. Details on the app can be found in Multimedia Appendix 1.

### Ethical Considerations

The dataset used in this research was collected by a service provider company for its own purposes. Legal consent for primary and secondary uses was obtained upon initial activation of the app. This retrospective study obtained no additional consent. All data were deidentified by the operating company (Wellmira Inc). The study protocol was approved by the Ethics Committee of the Kyoto University Graduate School and Faculty of Medicine (R4699).

### Materials

Analysis 1 included all registered users of the target app. We calculated the rate of active users as those who input any data into the app as a proportion of the number of registered users. In other words, active users hypothetically included people who did not drink on that day and those who do not habitually drink alcohol (including nondrinkers). The rate of users inputting alcohol data was calculated as those who input alcohol consumption data to the application as a proportion of the number of registered users.

For analysis 2, we used alcohol consumption records from 9991 randomly selected users. The method for selecting target users is described in the Data Exclusion subsection. Table 1 describes the background information of the users. To confirm the validity of the data, we compared individual alcohol consumption to the nationwide alcohol consumption records published by the National Tax Agency of Japan [10].

**Table 1:** Background information for 9991 randomly selected users of the target commercial smartphone app (CALO mama Plus [4]). We randomly selected these users from those who logged alcohol consumption records more than 20 times in a year for at least one calendar year from January 1, 2018, to December 31, 2022, and we confirmed that their age and alcohol input were logically reasonable in light of Japanese law and human physiology. Age represents age at the time of data extraction on December 31, 2022; weight, height, and BMI represent values at the time a user registered on the app. *P* values were calculated with the Brunner-Munzel test.

	Male	Female	*P* value	W statistic [11]
Participants, n (%)	6612 (66.2%)	3379 (33.8%)	—^a^	—^a^
Age (years), mean (SD)	52.9 (9.58)	50.1 (10.5)	<.001	12.8
Weight (kg), mean (SD)	73.0 (10.9)	57.0 (9.11)	<.001	–104
Height (m), mean (SD)	1.72 (0.0586)	1.59 (0.0519)	<.001	–214
BMI (kg/m^2^), mean (SD)	24.8 (3.32)	22.6 (3.36)	<.001	–60.9

^a^Not applicable.

### Analytical Conditions in Analysis 2

#### Data Exclusion

Initially, we randomly extracted 10,000 users who logged alcohol consumption data more than 20 times in a year for at least one calendar year from January 1, 2018, to December 31, 2022.

Considering the legal drinking age in Japan (20 years) and the average life expectancy in Japan, we excluded 7 users whose ages were 2, 3, 4, 6, 9, 18, and 123 at the time of data extraction (ie, December 31, 2022). In addition, we excluded 2 users who recorded unrealistic alcohol amounts (eg, over 1000 g net alcohol/day). Thus, the final number of target users was 9991.

#### Notification Evaluation

In the effectiveness evaluation of the notifications in analysis 2, the app logs for net alcohol consumption and alcohol-related notifications were analyzed to investigate whether notifications influenced drinking behavior following the notification.

We focused on 2 alcohol-related notifications derived from net alcohol intake: “good” (<20 g/day) and “bad” (≥60 g/day). We considered a notification effective when either of two conditions was satisfied: (1) at least one “no drinking day” occurred within 3 days, or (2) there were no days with ≥60 g/day within 3 days. The effectiveness rate was calculated by dividing effective notifications by the total number of notifications and multiplying by 100.

## Results

### Analysis 1

**Figure 1:**
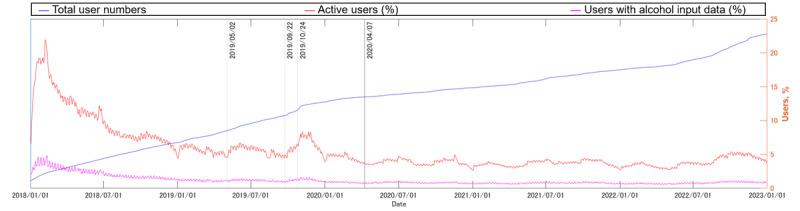
Trends among users of the target commercial smartphone app (CALO mama Plus [4]) with regard to the number of registered users, rate of active users, and rate of users with alcohol input data for the target period (ie, from January 1, 2018, to December 31, 2022). The solid black line indicates the date of the first state of emergency in Japan, starting on April 7, 2020, in key prefectures (Tokyo, Kanagawa, Saitama, Chiba, Osaka, Hyogo, and Fukuoka), which then expanded nationwide from April 16, 2020 [12]. The gray lines indicate the date of media exposure on TV programs. Although the exact number of users is undisclosed due to company restrictions, registered users increased 20-fold during the target 5 years. However, the rate of active users and users with alcohol input data remained stable over this period, with average rates of 5.6% (SD 3.19%) and 1.17% (SD 0.689%), respectively.

**Figure 2:**
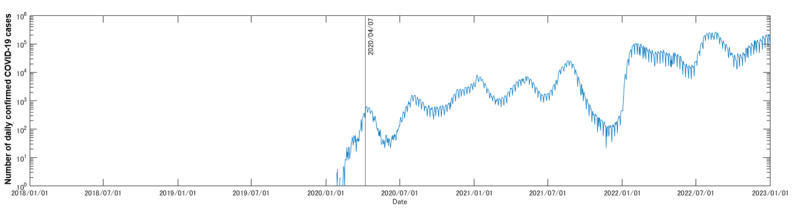
The number of daily confirmed COVID-19 cases in Japan [5] for the target period (ie, from January 1, 2018, to December 31, 2022). For easier understanding, we used logarithmic values in the y-axis. The solid black line indicates the date of the first state of emergency in Japan, starting on April 7, 2020, in key prefectures (Tokyo, Kanagawa, Saitama, Chiba, Osaka, Hyogo, and Fukuoka), which then expanded nationwide from April 16, 2020 [12]. We can confirm that this date was approximately the peak of the first wave of the COVID-19 outbreak in Japan.

Figure 1 shows the trend of the number of app users. Although the exact number is undisclosed due to company restrictions, registered users increased 20-fold during the target period. The rate of active users and users with alcohol input data remained stable over this period, with average rates of 5.6% (SD 3.19%) and 1.17% (SD 0.689%), respectively. Compared with the number of daily confirmed COVID-19 cases in Japan (Figure 2), no marked changes were confirmed in any of these values.

### Analysis 2

**Figure 3:**
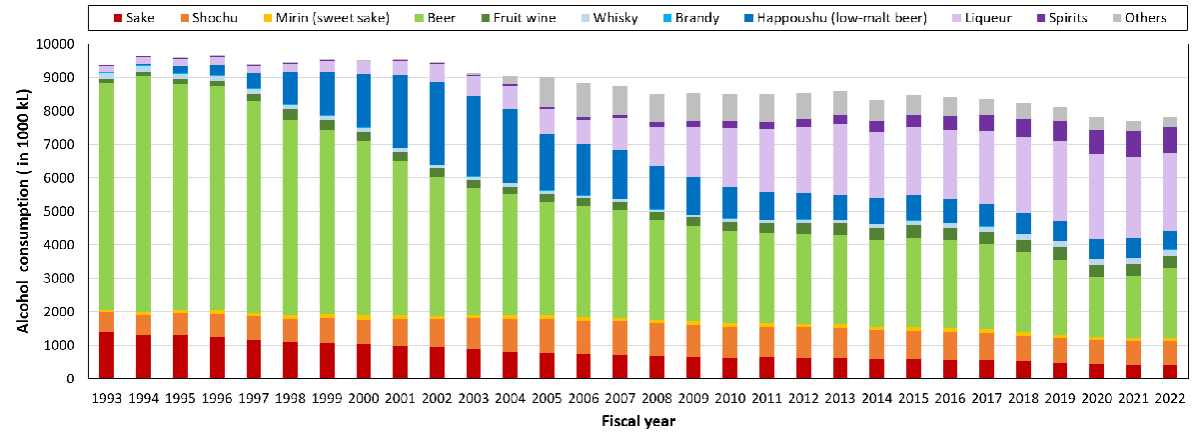
Alcohol consumption trends from the nationwide record published by the National Tax Agency of Japan [10]. These records are tallied according to the Japanese fiscal year (April 1 to March 31). “Mirin” (sweet sake) is not an alcoholic beverage, but a traditional Japanese sweet cooking wine containing approximately 10% alcohol. Overall, total alcohol consumption declined, with an annual drop of about 100,000 kL after 2018. Notably, there was a decrease of 300,000 kL between 2019 and 2020, and the amount of total alcohol consumption was similar in fiscal years 2020, 2021, and 2022.

Figure 3 shows Japan’s alcohol consumption trends, published by the National Tax Agency of Japan [10]. Overall, total alcohol consumption declined, with an annual drop of about 100,000 kL after 2018. Notably, there was a decrease of 300,000 kL between 2019 and 2020, and the total alcohol consumption was similar in fiscal years 2020, 2021, and 2022. Given that fiscal years 2020 and 2021 included the state of emergency due to the COVID-19 outbreak [12], this gap may reflect the government’s request to limit social gatherings.

**Figure 4:**
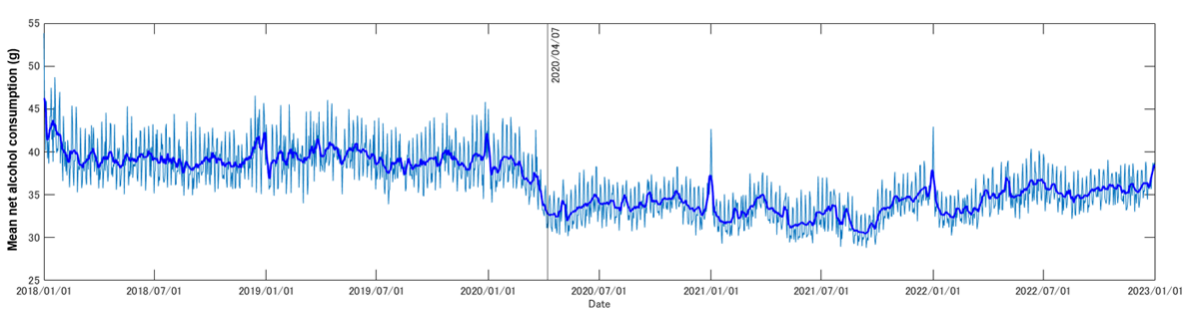
Mean net alcohol consumption obtained from the logs of the target commercial smartphone app (CALO mama Plus [4]) for the target period (January 1, 2018, to December 31, 2022). The bold blue line indicates the 7-day moving average. The solid black line indicates the date of the first state of emergency in Japan, starting on April 7, 2020, in key prefectures (Tokyo, Kanagawa, Saitama, Chiba, Osaka, Hyogo, and Fukuoka), which then expanded nationwide from April 16, 2020 [12]. Around the date of the first state of emergency in Japan, the data confirm a rapid decline. Aside from this, in all target years, the data confirm temporal peaks around the year end and the New Year holiday, which can be related to the increase in drinking parties at such times.

**Figure 5:**
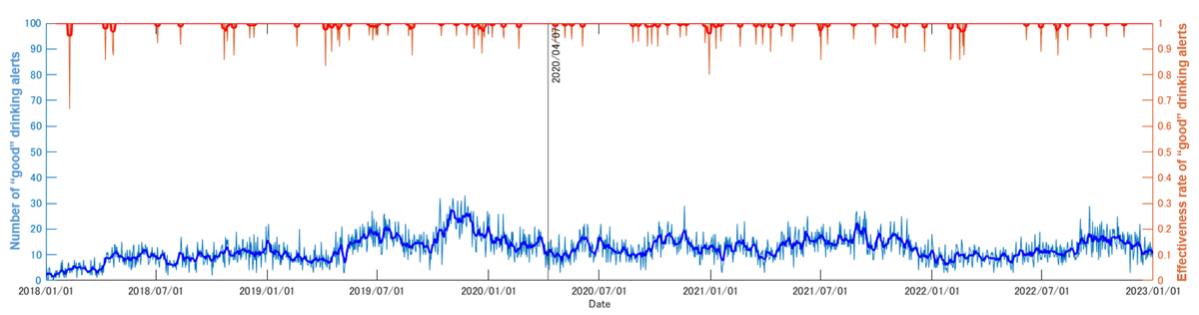
Number of “good” notifications, together with their effectiveness, obtained from the logs of the target commercial smartphone app (CALO mama Plus [4]) for the target period (January 1, 2018, to December 31, 2022). The pale blue line indicates the number of “good” notifications, whereas the pale red line indicates the effectiveness rate. The bold lines indicate the 7-day moving averages. The solid black line indicates the date of the first state of emergency in Japan, starting on April 7, 2020, in key prefectures (Tokyo, Kanagawa, Saitama, Chiba, Osaka, Hyogo, and Fukuoka), which then expanded nationwide from April 16, 2020 [12]. Unlike “bad” notifications, there were no marked changes around the date of the first state of emergency in Japan in either the number of notifications or their effectiveness.

**Figure 6:**
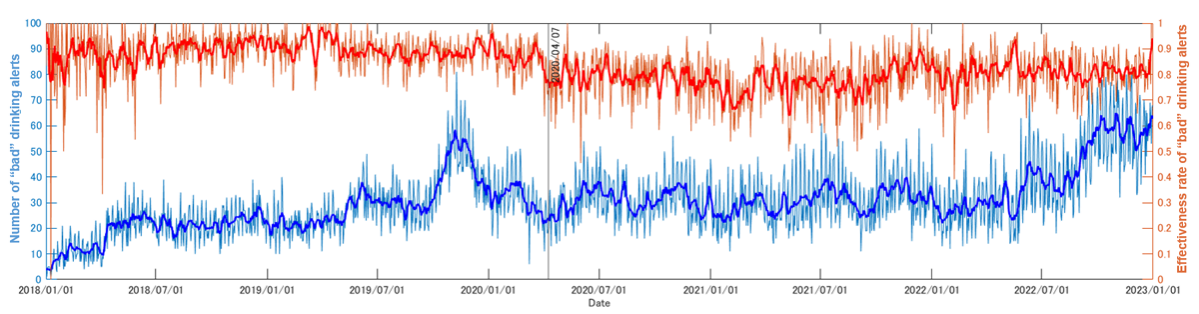
Number of “bad” notifications and their effectiveness, obtained from the logs of the target commercial smartphone app (CALO mama Plus [4]) for the target period (ie, from January 1, 2018, to December 31, 2022). The pale blue line indicates the number of “bad” notifications, whereas the pale red line indicates their effectiveness rate. The bold lines indicates the 7-day moving averages. The solid black line indicates the date of the first state of emergency in Japan starting on April 7, 2020, in key prefectures (Tokyo, Kanagawa, Saitama, Chiba, Osaka, Hyogo, and Fukuoka), which then expanded nationwide from April 16, 2020 [12]. We can confirm that there was a rapid decline in the effectiveness of the notifications near the date of the first state of emergency in Japan, by approximately 10%.

Figure 4 shows the trend in mean net alcohol consumption. Figure 5 shows the number and effectiveness of “good” notifications, whereas Figure 6 shows that of “bad” notifications. Notably, both mean net alcohol consumption (Figure 4) and the effect of “bad” alcohol-related notifications (Figure 6) decreased at the beginning of April 2020. Based on the Brunner-Munzel test, in the periods from January 1, 2018, to March 31, 2020, and April 1, 2020, to December 31, 2022, these differences were confirmed as significant (net alcohol consumption: W statistic=90.8; *P*<.001; effect of “bad” alcohol-related notifications: W statistic=34.4; *P*<.001). The Brunner-Munzel test results for other factors are shown in Figures 4-6, and other details can be found in Multimedia Appendix 1.

**Figure 7:**
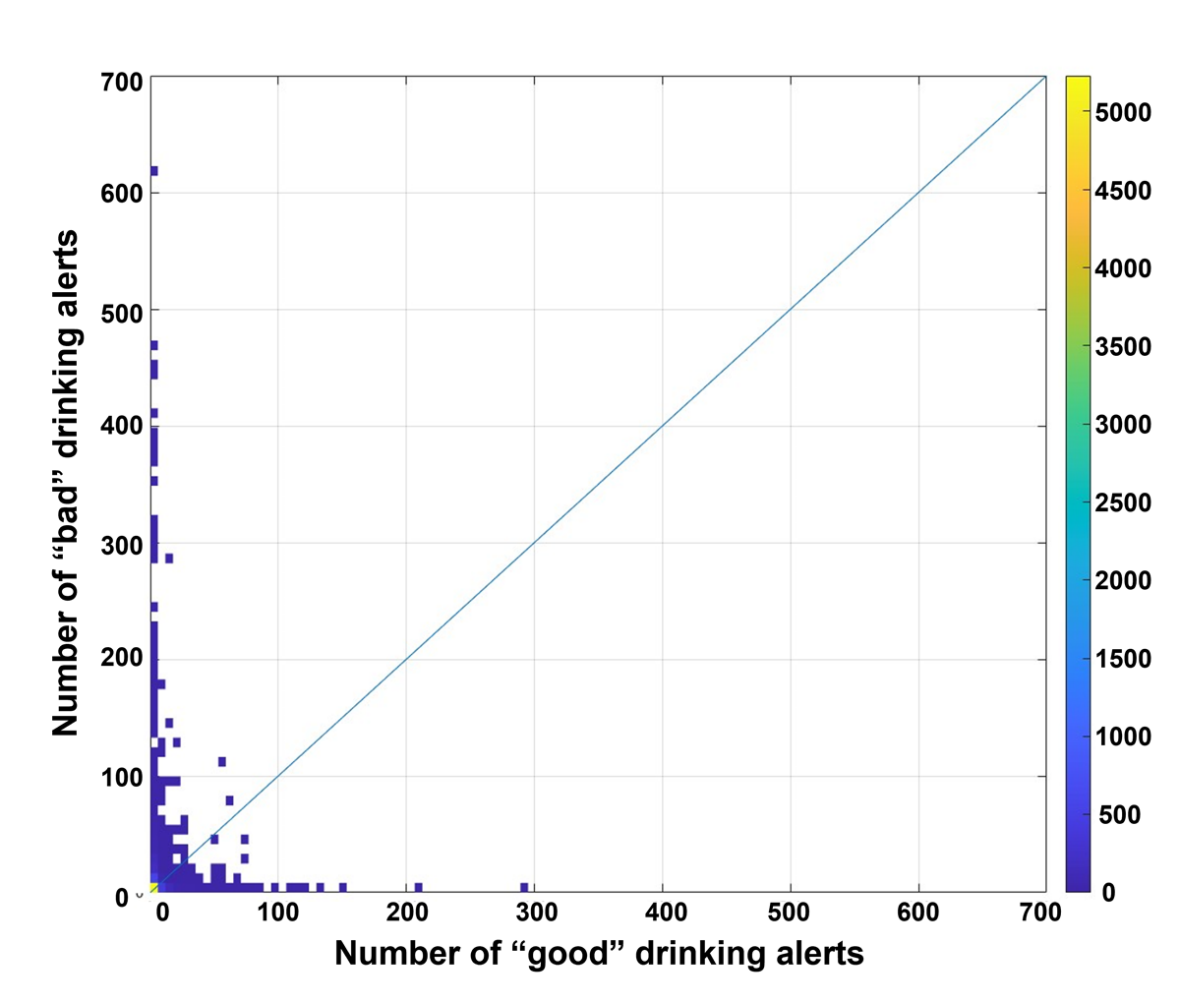
2D histogram of “good” drinking notifications and “bad” drinking notifications obtained from the logs of the target commercial smartphone app (CALO mama Plus [4]) for the target period (January 1, 2018, to December 31, 2022). The blue line indicates x = y, namely, that there were equal numbers of “good” and “bad” drinking notifications. The dots in the graph below the x = y line indicate the people who received more “good” notifications than “bad” and vice versa. Regarding the target users in our study, more people tended to have more “bad” notifications than “good” ones (ie, the dots in the area of the graph above the x = y line).

Comparing “good” (Figure 5) and “bad” (Figure 6) notifications, the number of “bad” notifications was roughly double. A 2D histogram (Figure 7) depicts the balance between “good” (x-axis) and “bad” notifications (y-axis) for each individual. This also confirms that “bad” notifications were more common. Accordingly, target users (n=9991) were classified into four groups: (1) more “bad” than “good” (n=4180, 41.8%); (2) equal (n=369, 3.69%); (3) more “good” than “bad” (n=2787, 27.9%); and (4) no notifications (n=2655, 26.6%). In groups 1, 2, and 3, a total of 6122 (61.3%) users had <10 “bad” notifications; thus, 8777 (87.8%) of all target users had no or <10 “bad” notifications. Among the remaining 1214 (12.2%) users, 1176 (96.9%) were classified as being in group 1 (more “bad” than “good”). Thus, only specific users, such as heavy drinkers, possibly tended to receive “bad” notifications.

## Discussion

### Summary of Findings

We confirmed that there was a decrease in net alcohol consumption in both government-provided data (Figure 3) and individual real-world data (Figure 4) that continued after 2020, which may have been related to the outbreak of COVID-19 and the state of emergency in Japan. Regarding the effect of the digital interventions, only the “bad” notifications showed a decreased effect near the first state of emergency. Taken together, this suggests that people who only consume alcohol at social gatherings may have reduced their consumption, whereas those who originally had no interest in “bad” notifications continued their consumption.

Compared with government-provided data, the individual real-world data showed more details on alcohol consumption behavior. In all target years, we confirmed temporal peaks in mean net alcohol consumption around the year end and the New Year holiday, which might be related to the increase in drinking parties at these times. This may paradoxically support our hypothesis that the request for restricting social gatherings led to reduced alcohol consumption in Japan.

### Limitations

The main limitations of this study are its generalizability and reliability. As we showed in Figure 1, the 5-year rates of active app users and users who input alcohol data were both lower than 10%. This low active user rate is one of the peculiarities of real-world data, indicating that our results represent only one aspect of reality.

Another limitation is the app specifications (detailed in Multimedia Appendix 1). Comparing alcohol consumption and notification records, we found “good” notifications in only 4.61% of records meeting the criterion of <20 g net alcohol/day, whereas “bad” notifications were found in 27.5% of records meeting the criterion of ≥60 g net alcohol/day. Therefore, our notification analysis can be considered a pilot study, and the efficacy of notifications should be assessed independently in a prospective study with modified alert systems.

The final limitation is the influence of the COVID-19 outbreak. Because almost half of our target period included the COVID-19 outbreak, our analyses may be affected by various socioeconomic factors. Further investigation focusing on individual behavior change is required to elucidate detailed relationships.

### Conclusions

A 5-year analysis of individual real-world data, including data from the COVID-19 outbreak period, showed that real-world data may reflect a more detailed reality than government-provided data.

## Appendix

Multimedia Appendix 1 Supplementary remarks and analysis.

## Data Availability

The real-world datasets on alcohol consumption analyzed during this study are not available as they are confidential business data of Wellmira Inc, but they are available from the corresponding author on reasonable request. The datasets for the number of daily confirmed COVID-19 cases in Japan are available from the Ministry of Health, Labor and Welfare in Japan [5], whereas the datasets for alcohol consumption trends are available from the National Tax Agency of Japan [10].
