# Supplementary material for: Real-World Data on Alcohol Consumption Behavior Among Smartphone Health Care App Users in Japan: Retrospective Study
Source: Online J Public Health Inform. 2025 Mar 25;17:e57084. doi: 10.2196/57084 (PMC11979541; doi:10.2196/57084)
Supplement: Multimedia Appendix 1 [file ojphi_v17i1e57084_app1.docx]

## Supplementary Material

### Remarks on Notification Alert and Application Settings

In *CALO mama Plus*, we can select any of four courses (ie, healthy diet, health maintenance, metabolic syndrome improvement, and low-carbohydrate (LOCABO)) and receive designated advice as a daily summary message depending on the selected course and daily lifelog data.

According to the application specifications, both “good” and “bad” notification alerting is determined based on the courses selected in the application and their lifelog data. Thus, not every user who met the notification criteria necessarily received “good” or “bad” notifications, which may vary daily based on their lifelog data even for the same user.

### Supplementary Analysis of the Brunner–Munzel Test on Actual Alcohol Consumption

We conducted the Brunner–Munzel test between January 1, 2018–December 31, 2020 and January 1, 2020–December 31, 2022 to confirm the difference that may stem from the COVID-19 outbreak in Japan on five values related to the actual alcohol consumption: mean net alcohol consumption (Figure 4), number of “good” drinking notifications (Figure 5, blue), the effectiveness of “good” drinking notifications (Figure 5, red), number of “bad” drinking notifications (Figure 6, blue), and the effectiveness of “bad” drinking notifications (Figure 6, red).

Except for the effectiveness of “good” drinking notifications (W statistic = 0.110, P = 0.91), a significant difference was confirmed in the mean net alcohol consumption (W statistic = 90.793, P<.001), the number of “good drinking notifications (W statistic = –5.34, P<.001), the number of “bad” drinking notifications (W statistic = –16.4, P<.001), and the effectiveness of “bad” alcohol-related notifications (W statistic = 34.418, P<.001).

### Remarks on the Relationship Between the Active Users’ Rate and the Number of “good” or “bad” Notifications

Unlike the mean net alcohol consumption (Figure 4), we could not confirm periodical or drastic changes in the number of “good” or “bad” notifications (Figures 5 and 6), except for temporal gradual increases around the autumn (ie, September–January) of 2019 and 2022. Both increases seem to be correlated with the active user rate (Figure 1) confirmed in these seasons.
